# INFOGEST 2.0 Protocol Applied to Animal-Derived Milk and Dairy Products: A Systematic Review of Six Years of Scientific Effort

**DOI:** 10.3390/foods15111871

**Published:** 2026-05-25

**Authors:** Giuseppe De Santis, Olubukunmi Amos Ilori, Diana Marisol Abrego-Guandique, Pierluigi Plastina, Paola Tucci, Erika Cione

**Affiliations:** Department of Pharmacy, Health and Nutritional Sciences, University of Calabria, 87036 Rende, Italy; dsngpp99t11b774s@studenti.unical.it (G.D.S.); olubukunmiamos.ilori@unical.it (O.A.I.); dianamarisol.abregoguandique@unicz.it (D.M.A.-G.); pierluigi.plastina@unical.it (P.P.); paola.tucci@unical.it (P.T.)

**Keywords:** INFOGEST, milk, dairy products, in vitro digestion, bioaccessibility

## Abstract

The INFOGEST protocol is a standardised in vitro digestion model widely utilised to evaluate the digestibility and bioaccessibility of nutrients in diverse food matrices. This review focuses on its application since 2020 (after the publication of the INFOGEST 2.0 model) to milk and dairy products, which often serve as a suitable food matrix in digestion studies. By analysing 50 studies selected using a semi-automated method, this review highlights its strong performance in reproducing general digestive trends, including peptide fingerprint profiling, consistent high-protein digestibility, and matrix-dependent lipid and mineral bioaccessibility. The model is particularly effective in evaluating structural modifications of dairy products and their impact on digestive behaviour. However, its application remains skewed toward bovine systems, limiting broader relevance to other dairy matrices. Methodological variability, including protocol modifications and emerging semi-dynamic adaptations, poses challenges to reproducibility. Furthermore, reliance on simplified downstream models constrains the physiological interpretation of bioactivity and nutrient absorption. Future progress requires harmonised dynamic extensions, expanded use of advanced biological systems, and inclusion of diverse dairy matrices. Collectively, these advances will support a shift from descriptive bioaccessibility toward more predictive assessments of nutrient bioavailability. This six-year, non-topic-dependent bibliometric analysis contextualises the expanding adoption of INFOGEST 2.0 as reflected in its versatility and evolving scope, positioning it as a cornerstone tool for advancing our understanding of dairy nutrition, digestion-derived bioactivity, and ultimately, the relationship between dairy consumption and human health.

## 1. Introduction

**Figure 1:**
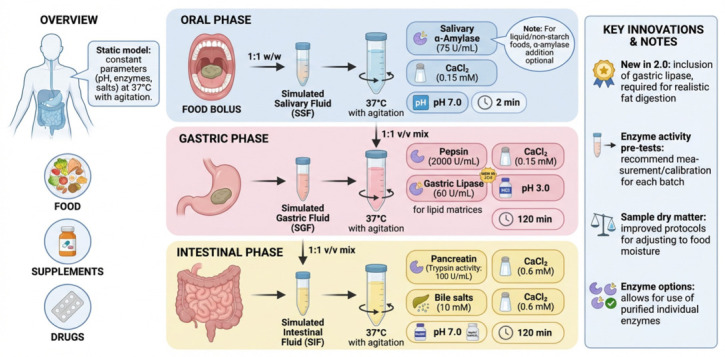
INFOGEST protocol. Created with FigureLabs.

In recent decades, scientific research in the field of food and nutrition has made considerable progress, taking full advantage of the development of new methodologies for the study of in vitro digestion. The concept of in vitro gastrointestinal digestion, also called digestomics, is a technique used to simulate and study the process of digestion of food outside the human body under controlled conditions [1]. This method reproduces the biochemical, enzymatic and physical conditions of the human gastrointestinal tract, allowing for the observation and analysis of food and nutrient degradation and transformation during digestion [2,3]. There are two types of in vitro digestion that are commonly used: static and dynamic methods. Static models are much less complex and easier to set up. Hence, the static digestomic protocol was proposed in 2014 by the COST Action called INFOGEST for the static model. This involved a network of multidisciplinary experts from over 30 countries developing a static digestion method, modelled after a healthy adult digestion apparatus, with international consensus to harmonise experimental conditions. Following widespread adoption, increased enzyme availability, and a need to better understand the degradation of certain food components, the model was further standardised (as INFOGEST 2.0) in 2019 to include, amongst others, the oral phase and gastric lipase [2,3]. The INFOGEST 2.0 model is a static digestion technique that maintains a constant pH at each stage of the digestion and a constant ratio between the meal and the digestive fluid. The digestion process is divided into three phases: oral, gastric, and intestinal (Figure 1). This allows for the evaluation of food digestion endpoints while avoiding the use of laboratory animals. It also permits the examination of the products of food macromolecules as a result of digestion (fatty acids, simple sugars, peptides/amino acids, etc.) [3], the study of micronutrient bioaccessibility [4,5], and the determination of the digestive stability of different food bioactive components, such as microRNAs [6] and genetically modified plant products [7]. Milk is an important dietary source of essential vitamins and minerals, high-quality proteins, and fats, and it is a sustainable and complete food [8,9]. The relationship between milk fat and health has been widely investigated [10,11]. Although healthy people suffer from milk-related gastrointestinal discomfort despite not being lactose intolerant, the mechanisms behind such conditions are still unknown [12]. The interaction of milk during digestion is thus of great importance in providing tools towards a better understanding of the effects of milk on health [13]. While previous reviews have broadly addressed the INFOGEST protocol for the digestion of numerous food groups, the dairy group has not been critically examined [1,14], and none have systematically focused exclusively on the updated INFOGEST framework applied to the full range of milk and dairy products. Moreover, earlier works did not integrate a systematic review of studies covering both macro- and micronutrient bioaccessibility. Considering that milk is usually employed as a validation matrix for this protocol and given the potential variations in protocol adaptations that usually follow the harmonisation of a model [15,16], we aimed to give a coherent understanding of the extended dairy digestion within the INFOGEST framework following the publication of the INFOGEST 2.0 model recommendation.

## 2. Materials and Methods

### 2.1. Systematic Literature Review

The systematic review was conducted according to the Preferred Reporting Items for Systematic Reviews and Meta-Analyses (PRISMA) guidelines [17]. The methodological approach is based on three steps: paper location and selection, paper analysis, and results presentation. We adopted a semi-automated approach using the MySLR platform upon registration (available at https://myslr.unical.it, accessed on 6 January 2026). This digital tool reproduces “human-like intelligence” as closely as possible by implementing the LDA algorithm. We loaded the papers on MySLR to offer a complete and exhaustive overview of scientific research [18]. The MySLR platform was already used previously [19,20]. The MySLR platform also performed an initial automated deduplication and exclusion of non-original articles (reviews, editorials, conference papers, letters) using a pre-trained classifier based on publication type metadata.

### 2.2. Paper Location, Selection, and Data Extraction

Two investigators (G.D.S. and D.M.A-G.) independently conducted systematic searches on PubMed, Scopus, and Web of Science Core Collection on 15 January 2026. The search was limited to peer-reviewed original articles published or accepted between 1 January 2020 and 31 December 2025 (inclusive). No language restrictions were applied during the search, but non-English articles were later excluded at the full-text stage (see exclusion criteria). The following search string was used for PubMed (adapted for Scopus and Web of Science with appropriate field tags): (infogest[Title/Abstract] OR “INFOGEST 2.0”[Title/Abstract] OR “INFOGEST protocol”[Title/Abstract]) AND (digestion[Title/Abstract] OR “in vitro digestion”[Title/Abstract] OR digestibility[Title/Abstract] OR bioaccessibility[Title/Abstract]) AND (dairy[Title/Abstract] OR milk[Title/Abstract] OR cheese[Title/Abstract] OR yoghurt[Title/Abstract] OR yogurt[Title/Abstract] OR whey[Title/Abstract] OR casein[Title/Abstract] OR “fermented milk”[Title/Abstract]). For Scopus, the same terms were applied using the TITLE-ABS-KEY field codes. For Web of Science, TS = (topic search) was used.

After retrieval, all records were exported to the MySLR platform. Duplicates were identified and removed using an exact match algorithm based on the following fields: title, authors (surname + initials), journal, volume, issue, page range, DOI, and publication year. Records were considered duplicates if all these fields matched exactly. In cases of partial mismatch (e.g., different DOI but identical title and authorship), manual verification was performed by two independent reviewers (G.D.S. and D.M.A-G.).

For this systematic review, dairy products are operationally defined as foods derived exclusively from the milk of lactating animals. This includes, but is not limited to, bovine (cow), goat, sheep, buffalo, mare, and camel. The following forms are included: liquid milk (raw, pasteurised, UHT, skimmed, whole, powdered), fermented milks (yoghurt, sour cream, kefir, cultured buttermilk), cheeses (fresh, soft, hard, processed, mould-ripened), whey and whey derivatives (sweet whey, acid whey, whey protein isolate/concentrate), cream, butter, ghee, and infant formula based on animal milk. This definition explicitly excludes plant-based milk alternatives and dairy–plant hybrid products. Inclusion criteria were defined as follows: (i) original research articles applying the INFOGEST protocol; (ii) studies in which the food matrix tested was a milk or dairy product (including bovine, goat, sheep, buffalo, mare, as well as derived products such as cheese, yoghurt, whey, cream, fermented milks, or dairy-based emulsions/gels); (iii) articles reporting sufficient information on the type of milk/dairy matrix and the analytical techniques used post-digestion; (iv) full-text articles written in English. Exclusion criteria were: (i) reviews, meta-analyses, letters, conference papers, comments, book chapters; (ii) studies not including certain updates from the 2019 version; (iii) studies not involving any dairy product (e.g., plant-based milks without presence to dairy, or non-dairy protein sources); (iv) studies that did not perform actual in vitro digestion (e.g., only enzyme assays or theoretical simulations); (v) articles where the full text was not available in English; (vi) duplicate publications from the same dataset. Plant-based milk alternatives were excluded unless used as comparators to dairy milk. This avoids confounding factors (different protein structures, antinutritional factors, dietary fibre) that are not representative of animal-derived dairy matrices and would hinder direct comparison across studies.

Disagreements were resolved through discussion to reach a consensus and, in some cases, by means of a third reviewer (P.P.). Data from all included articles were extracted by two authors (G.D.S. and D.M.A-G.) and checked by two authors (E.C. and P.T.). The following information was recorded: authors’ names, publication year, study country, type of milk, thematic area, and methodology used post-INFOGEST, as well as results.

## 3. Results

### 3.1. Qualitative Analysis of INFOGEST Usage on Dairy Products

**Figure 2:**
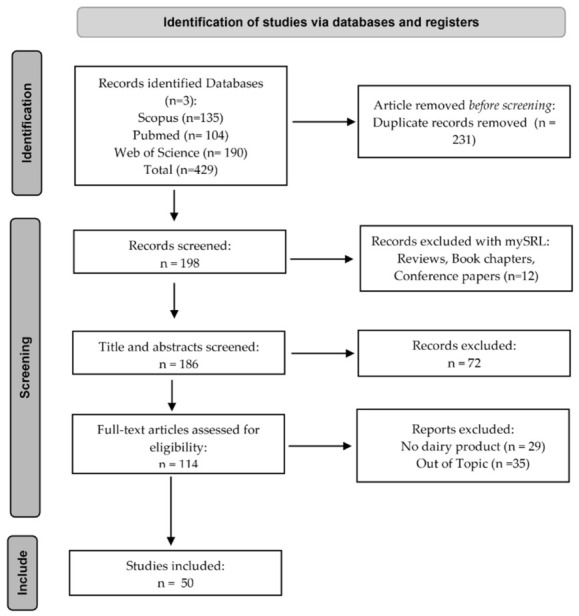
PRISMA flow diagram showing the algorithm of selection of eligible studies included on MySLR.

A total of 429 records were identified from the initial literature search of the three databases (PubMed, Scopus, and Web of Science), of which 231 were duplicates. Of these, the MySLR tool removed 12 papers because they were reviews. Of the 186 studies remaining, 72 were discarded based on their abstracts. After full-text reading and analysis, 64 records were further excluded for failing to meet the inclusion/exclusion criteria. Therefore, a total of 50 studies were considered eligible. The PRISMA flowchart in Figure 2 shows the selection of the studies for this systematic review.

Overall, 50 studies published between 2020 and 2025 were included in the systematic review (Table 1). The selected literature primarily falls within the domains of Food Science and Nutrition, with additional contributions from Biochemistry, Microbiology, Biological, Food Engineering, and Agricultural Sciences, among others. Geographically, the studies were predominantly conducted in Europe, with a majority of contributions from France, Italy, Portugal, Spain, Denmark, and Switzerland, followed by Brazil, China, Greece, Ireland, the UK, and the USA, indicating a strong European research focus alongside a growing global interest.

From a methodological perspective, the included studies displayed a high degree of analytical heterogeneity, with a clear predominance of chromatographic and mass spectrometry-based techniques. In particular, LC-MS/MS, UHPLC-HRMS/Q-Orbitrap, GC-MS, and GC-FID were the most frequently employed platforms for molecular characterisation of proteins, peptides, lipids, fatty acids, metabolites, and micronutrients. Protein separation techniques, especially SDS-PAGE, often combined with mass spectrometry approaches (e.g., MALDI-TOF/TOF, ESI-MS), were widely used in studies focused on protein and peptide profiling. Several investigations integrated compositional analyses with biochemical and functional assays, including antioxidant capacity tests, total phenolic and flavonoid content determination, and semi-automated gastrointestinal digestion models (e.g., pH-stat-based approaches). Notably, a substantial proportion of studies published from 2022 onwards incorporated in vitro cellular models, frequently combined with gene expression analyses (qPCR), cell viability assays, inflammatory markers, and oxidative stress measurements. In parallel, multiple studies focused on mineral and trace element characterisation, employing FAAS, ICP-OES, and ICP-MS, often supported by multivariate statistical analyses such as principal component analysis (PCA). Additionally, a subset of studies adopted advanced structural, colloidal, and physicochemical characterisation techniques, including NMR, FTIR-ATR, CLSM, AFM, NTA, tensiometry, rheology, and particle-size/zeta-potential measurements, to investigate food matrix organisation and digestion-related transformations.

Bovine milk—in whole, skimmed, pasteurised, UHT, organic, or genotype-specific forms (e.g., A1/A2 β-casein)—was the primary food matrix in the majority of studies, reflecting its central role as a reference model in dairy and nutrition research (Table 1). Several studies focused on isolated bovine milk components, including whey protein isolate (WPI), whey protein concentrate (WPC), lactoferrin, and milk fat globules, as well as engineered dairy-like systems (e.g., protein-stabilised emulsions, polysaccharide-structured gels, and nano-carrier formulations). A substantial proportion of studies investigated fermented dairy products, including yoghurt, fermented milks, sour cream, skyr, cream cheese, gouda-type cheese, cheddar, minas frescal, and fresh cheeses, often differing in fat content, protein composition, starter cultures, or fermentation technology.

The food matrices investigated across the included studies are summarised in Table 1. Beyond bovine milk, non-cow dairy sources are also represented. These included goat, sheep, buffalo, mare, and human milk, either as whole matrices or as derived products (e.g., whey, extracellular vesicles, or milk fractions). Goat and sheep milk were particularly frequent in comparative studies, while buffalo milk and mare milk were examined in more targeted investigations addressing unique compositional or functional traits. Human milk and infant formula were included in a limited number of studies, mainly for comparative or translational purposes.

Several studies focused specifically on whey and whey-derived products, including sweet whey from cheese production, acid whey, protein isolates, and whey-based delivery systems, underscoring the relevance of whey as both a by-product and a functional ingredient. Additionally, a smaller subset of studies examined complex or reformulated dairy systems, such as fortified yoghurts, lipid-enriched products, nanoemulsions, and bioactive-compound-enriched products (e.g., ω-3 fatty acids, α-tocopherol, milk fat globule membrane).

Table 2 summarises the macronutrients investigated using the INFOGEST 2.0 simulated gastrointestinal digestion protocol and the primary outcomes assessed across the included studies. Proteins were the most frequently studied macronutrient, either as whole milk proteins, purified fractions (e.g., κ-casein, lactoferrin), whey protein isolates, or protein hydrolysates. Across studies, protein-related outcomes predominantly included digestibility, bioaccessibility, and peptide release following digestion. Digestibility was commonly assessed through degree of hydrolysis, proteolysis kinetics, and release of free amino acids, whereas peptide release studies focused on identifying digestion-derived peptides, including low-molecular-weight and digestion-resistant fractions. Several investigations extended these analyses to functional or biological endpoints, such as effects on glucose homeostasis markers or immunomodulatory activity of bioactive peptides generated during digestion. A smaller but consistent subset of studies addressed lipid digestion, focusing on lipid digestibility, free-fatty-acid (FFA) release kinetics, and bioaccessibility of lipid classes, including phospholipids and short- and medium-chain triglycerides. These studies often applied pH-stat approaches and kinetic modelling to characterise lipolysis under simulated gastrointestinal conditions. More recent works integrated lipid digestion outcomes with structural and physicochemical parameters of the food matrix, highlighting the influence of matrix organisation on lipid bioaccessibility.

Carbohydrates were less frequently investigated and were primarily represented by studies on exopolysaccharides (EPSs) and prebiotic components. These works focused on EPS quantification during fermentation, stability under digestive conditions, and their relationship with functional properties, such as postbiotic activity or sensitivity to pH and temperature. Several studies adopted a multi-macronutrient approach, simultaneously evaluating proteins, lipids, and/or carbohydrates within the same food matrix. These integrated designs enabled comparative assessment of digestion behaviour and bioaccessibility across macronutrient classes and supported a more holistic understanding of food matrix disassembly during digestion.

Overall, the evidence base of micronutrient post-digestion studies in animal dairy products is dominated by mineral bioaccessibility, with a smaller but growing number of investigations addressing fat-soluble vitamins, carotenoids, bioactive lipids, and other non-classical micronutrients (Table 3). Minerals represent the most frequently studied micronutrient class. Calcium was the most extensively investigated element, either alone or in combination with other minerals (e.g., magnesium, phosphorus, potassium, zinc), with outcomes primarily focused on bioaccessibility, or solubility following digestion. Several studies extended mineral analysis to multi-element panels, including both essential trace elements (e.g., iron, zinc, selenium, copper, manganese) and non-essential or potentially toxic elements, evaluated mainly in terms of bioaccessibility. Comparative designs assessing different food matrices, including dairy versus plant-based systems, were also reported.

A second group of studies focused on fat-soluble micronutrients, including vitamin K, α-tocopherol (vitamin E), and provitamin A carotenoids (β-carotene). These investigations primarily assessed bioaccessibility, stability, and recovery after in vitro digestion, often highlighting the influence of food matrix composition, lipid content, and processing conditions (e.g., encapsulation, pH, temperature) on micronutrient release and retention. Several studies addressed bioactive lipids, particularly ω-3 fatty acids, evaluating their bioaccessibility, digestion kinetics, and contribution to antioxidant capacity within complex dairy matrices. These works often integrated kinetic lipolysis data with bioaccessibility endpoints, providing insight into the digestion behaviour of lipid-soluble micronutrients.

Finally, more recent investigations expanded the scope of INFOGEST applications to include non-traditional micronutrients and bioactive compounds, such as phenolics, flavonoids, conserved microRNAs, and milk extracellular vesicles (EVs). These studies primarily focused on stability, integrity, recovery, and matrix-dependent retention during digestion, reflecting an emerging interest in the fate of regulatory and functional food components beyond classical vitamins and minerals.

### 3.2. Demonstrated Biological Activities of Dairy Digesta in In Vitro Systems

**Figure 3:**
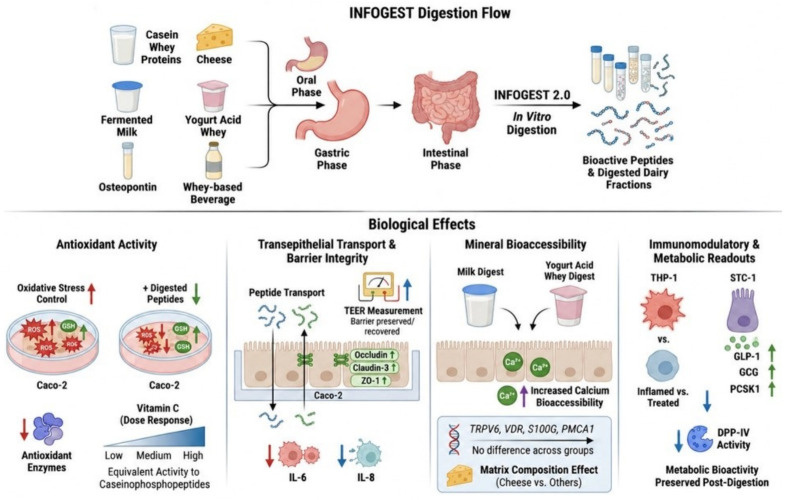
Cellular effects of digested dairy matrices using the INFOGEST 2.0 Protocol.

Only eight studies among the selected studies complement the standardised INFOGEST digestion protocol with intestinal or intestinal-related cell models (Table 4 and Figure 3). Most studies employed Caco-2 monolayers or Caco-2/HT-29 co-cultures, while selected investigations extended the analysis to NCM460 healthy intestinal epithelial cells or extra-intestinal models (e.g., Saos-2 osteoblast-like cells). The food matrices investigated included casein and whey proteins, fermented milks, acid whey from yoghurt, caseinophosphopeptides, osteopontin, and whey-based beverages. However, the focus was on bioactive peptides.

#### 3.2.1. Antioxidant Activity and Oxidative Stress Modulation

Several studies consistently reported that digestion enhanced or preserved the antioxidant activity of dairy-derived matrices. Digestion-resistant whey peptides significantly reduced intracellular ROS levels in Caco-2 cells exposed to oxidative stress, while preventing glutathione depletion and excessive antioxidant enzyme activation [40]. Similarly, fermented milks enriched with exopolysaccharide-producing *Lactiplantibacillus plantarum* strains showed improved antioxidant capacity after digestion and attenuated ROS production in NCM460 cells under both oxidative and inflammatory conditions [71]. Casein-derived peptides and caseinophosphopeptides generated after INFOGEST digestion displayed dose-dependent antioxidant effects, comparable to or exceeding vitamin C in Caco-2/HT-29 co-cultures and retained activity after intestinal metabolism [21]. Whey-based beverages also showed increased antioxidant bioaccessibility post-digestion, with bioaccessible fractions significantly lowering ROS generation in Caco-2 cells [30].

#### 3.2.2. Transepithelial Transport and Barrier Integrity

Multiple studies evaluated intestinal transport and epithelial integrity following digestion. Whey peptides and milk-derived protein fragments exhibited measurable transepithelial transport across Caco-2 monolayers, with permeability influenced by peptide size, terminal residues, and resistance to brush-border peptidases [32]. TEER measurements confirmed preservation or recovery of barrier integrity following exposure to selected digested dairy matrices.

#### 3.2.3. Mineral Bioaccessibility and Cellular Uptake

Calcium bioavailability was specifically addressed in yoghurt acid whey and dairy matrices. Following INFOGEST digestion, calcium bioaccessibility was significantly higher in yoghurt acid whey compared with milk, although no differences were observed in calcium transport across Caco-2 cells or in the expression of calcium transport-related genes (TRPV6, VDR, S100G, PMCA1) [63]. Comparable results were reported for cheeses, where mineral bioaccessibility depended on matrix composition and processing, while cellular transport efficiency did not strictly mirror soluble mineral fractions, highlighting the importance of the food matrix rather than total mineral content alone [29].

#### 3.2.4. Immunomodulatory and Metabolic Readouts

Beyond antioxidant and barrier-related endpoints, digested whey fractions modulated inflammatory and metabolic pathways. Sweet whey-derived peptides altered the expression of inflammation-related genes in THP-1 macrophages, with effects depending on milk origin and inflammatory status [61]. In enteroendocrine models, digested matrix enhanced GLP-1 secretion, upregulated GCG and PCSK1, and inhibited DPP-IV activity after intestinal barrier passage, indicating preserved metabolic bioactivity after digestion [44].

## 4. Discussion

The INFOGEST protocol has emerged as a pivotal technique for studying in vitro gastrointestinal digestion, providing a standardised methodology for evaluating the digestibility and bioavailability of nutrients across diverse food matrices. This review highlights its application to milk and dairy products. Notably, the methodology’s impact has seen a significant rise since 2019, following the publication of the INFOGEST 2.0 protocol by Brodkorb and collaborators [3], which has driven widespread adoption and expanded its relevance in food and nutritional sciences. Several factors may culminate in the consideration of dairy products as food matrices of choice in the INFOGEST application. This includes, among others, the use of milk as the reference validation matrix by inter-laboratory trials of the initial INFOGEST procedure [15] and the demonstrated physiological relevance for milk digestion specifically, i.e., correspondence to in vivo outcomes [73]. Similarly, we observed a significant application of the digestion model to the food group in question in Europe and Latin America (mainly Brazil). This may be driven by the consistent use of dairy as food in these regions [74]. While a number of developing countries produce and consume considerable quantities of dairy products, especially buffalo milk [75,76], the INFOGEST application on such food matrices is very limited.

A clear convergence in analytical approaches was observed across studies, with recurring use of chromatographic, mass spectrometric, spectrophotometric, and imaging techniques. This consistency suggests the emergence of an implicit analytical framework accompanying standardised in vitro digestion models. High-resolution mass spectrometry platforms are predominantly employed to characterise the molecular composition of digesta, while chromatographic techniques facilitate fractionation and targeted analysis [77]. In parallel, spectrophotometric and electrophoretic methods remain essential for quantifying digestion kinetics and validating the extent of proteolysis. Targeted assays, such as ELISA and qPCR, further enable the tracking of specific biomolecules, reflecting increasing interest in the stability and bioactivity of functional components. Additionally, imaging approaches provide insight into microstructural transformations that influence digestive behaviour. Collectively, these techniques constitute a multi-scale analytical strategy spanning molecular, structural, and functional levels. This consistency in analytical approaches across studies suggests an implicit standardisation not only of digestion protocols but also of downstream characterisation, enabling comparability and reproducibility across the field.

### 4.1. Bovine: The Matrix of Interest

Bovine milk and its products, as the current trend suggests, remain the food matrix of interest, while other members of the dairy food group are barely studied. This includes buffalo milk, which accounts for 15% of the global dairy usage, being the second-highest contributor [78]. This food matrix bias reflects broader trends in dairy research, where cow-derived systems remain the default experimental model. Also, there is consideration for industrial relevance and compatibility as the primary validation matrix [14]. The extensive compositional characterisation and methodological familiarity associated with bovine systems likely contribute to their preferential selection, reinforcing a cycle of continued focus. However, this dominance limits the broader applicability and direct extrapolation of findings derived predominantly from bovine systems, as alternative dairy matrices, including buffalo and sheep milk, exhibit distinct physicochemical properties that influence digestion behaviour and nutrient bioaccessibility [79]. Also, while casein is the most studied component of the products in terms of hydrolysis and digestibility, whey proteins often find applications in studies examining modified food structural behaviour under digestion.

### 4.2. Dairy Protein Digestion Output

While a broadly conserved peptide fingerprint emerges at the end of digestion, this convergence reflects both a strength and a limitation of the approach. On the one hand, the digestive model has enabled systematic comparisons of digestibility and peptide release at the level of protein fractions and variants. The reproducibility of intestinal peptide profiles across static and dynamic systems and with reported correlations to in vivo data of up to 0.8 [27] supports the model’s robustness in capturing general proteolytic outcomes. Casein-derived peptides, particularly from κ- and β-casein, consistently dominate investigations [30,32,34,38,47], resulting in large repertoires (as high as 500) of peptides from resistant proteins such as caseinophosphopeptides [21,54]. On the other hand, this apparent uniformity may obscure physiologically relevant nuances. Variations introduced by calcium content (for instance, low calcium content consistently yields high peptide variety), casein structuring, or microbial diversity in fermented products demonstrate that upstream factors can significantly reshape peptide composition [21,22], yet these differences are not always fully resolved within standard INFOGEST conditions. Efforts to incorporate brush border enzymes or adapt the protocol to specific populations (e.g., elderly or preterm models) represent important advances [32], revealing extended cleavage patterns and distinct peptide pools.

Parallel progress has been made in understanding how protein structure and food matrix design influence digestion kinetics, an area where INFOGEST has proven particularly informative. Studies show that modifying protein architecture, through hydrolysis, gelation, high-pressure processing, or encapsulation, can significantly alter enzyme accessibility and shift the site and extent of proteolysis as well as the release of other dairy food components [25,34,38,44,72]. For example, whey protein hydrolysates are consistently more digestible than isolates, while gel microstructure and surface area dictate gastric breakdown rates [44,72]. Encapsulation and conjugation strategies further demonstrate controlled digestion, often delaying hydrolysis until the intestinal phase [48,56]. In spite of these modifications, the role of phenotypic and genotypic differences on dairy protein origin, particularly casein variants, does not go unnoticed [34,38,47]. These findings relate the model’s sensitivity to structural modifications and natural variability as well as its utility in designing functional foods.

Overall digestibility values are typically high (80–99%), aligning well with in vivo data and reinforcing confidence in quantitative outputs such as DIAAR estimates [45,80], and this stands as perhaps the most progressive application of the model. This may have potential applications in food composition research and industry, enabling estimation of food protein quality without the need for in vivo models. However, the model is less discriminative in linking these differences to downstream physiological effects. While numerous bioactive peptides are identified in dairy protein digesta—exhibiting antioxidant, antihypertensive, metabolic (e.g., GLP-1 stimulation, DPP-IV inhibition), and barrier-protective functions (Figure 3)—the translation of these findings remains constrained by the model’s lack of unified absorption, metabolism, and systemic feedback models that can directly fuse the demonstrated activity to a specific peptide in the dairy product digesta [21,30,40,44,61,81]. Additionally, dose-dependent or context-specific effects (including potential pro-inflammatory responses) are difficult to interpret within a purely digestive framework. Thus, while INFOGEST has significantly advanced the characterisation of dairy protein digestion and peptide bioactivity, its application, going forward, will benefit from sound experimentation that can complement models to fully resolve the functional relevance of the peptides it so effectively generates.

### 4.3. Dairy Lipid Digestive Behaviour

With the increasing availability of pH-stat instrumentation, its accessories, and its accompanying simplicity, which present an attractive avenue for studying lipase activity and lipolysis [82], the use of the in vitro digestion model to understand lipid digestion in dairy systems reflects a growing capacity to capture trends in fat bioaccessibility. A consistent observation is that dynamic and semi-dynamic models yield higher and more physiologically nuanced estimates of lipolysis and fatty acid release than static protocols, particularly for milk fat. For example, dynamic conditions enhance overall bioaccessibility, while also revealing preferential release of medium-chain fatty acids (C8:0–C12:0) over longer-chain species [23,26] and shifting lipid profiles toward increased relative proportions of MUFAs and PUFAs, with concomitant reductions in SFAs [30]. However, these outputs are strongly modulated by the surrounding food matrix: increasing matrix complexity can either enhance lipolysis (e.g., through protein-mediated stabilisation of fat globules) [36] or suppress it (e.g., via polyphenol-rich components such as tea or grape seed) [23,37], highlighting that the model is sensitive to compositional context but not always predictive across substrates. Hence, advances in structural and processing considerations have demonstrated the protocol’s utility. Homogenization, for instance, improves lipid digestibility and phospholipid retention [35]. Hence, the digestive model can detect microstructural effects on enzyme accessibility. Age-related adaptations (e.g., elderly models) equally suggest reductions in lipolysis in the presence of digestive-resistant protein or low gastric lipase, raising concerns of inefficient dairy fat digestion in children or infants with low human gastric lipase production, such as non-*Helicobacter pylori* gastritis [57,83]. At a broader level, the model has been applied to help profile post-digestion dairy lipids, providing a clearer understanding of the in vivo trend. This progress has benefited greatly from the semi-dynamic adaptations, allowing more thermal and pH control during the digestion process, although such outputs still require cautious interpretation, particularly based on the specific digestive conditions employed, since the INFOGEST recommendations are sometimes not strictly applied.

### 4.4. Micronutrient Bioaccessibility and Bioactive Components

Application of the model to calcium and other micronutrients in dairy systems demonstrates both its utility in estimating bioaccessibility and its limitations in predicting true bioavailability. Calcium bioaccessibility across dairy matrices is generally moderate but very variable (ranging from 11 to 75%) [29,43,49,51,63,70], often exceeding that of plant-based alternatives [43,70]. While the bioaccessibility of many minerals falls within this range, others might have a higher level than calcium within the same food matrix [43,51]. Also, variability in bioaccessibility may be dependent on dairy fraction or the nature of production (e.g., yoghurt acid whey vs. milk, or organic vs. conventional milk), although not usually on dairy origin [51,63]. Casein digestion plays a central mechanistic role, with the release of large numbers of caseinophosphopeptides likely contributing to mineral solubilisation and stabilisation during digestion, suggesting a higher potential of absorption in caseinophosphopeptide-rich dairy products such as cheese [21,29,84,85]. Despite these variations, cellular uptake may be comparable [50,63]. Fat-soluble vitamins also show substantial but more variable bioaccessibility [25,28,86]. Structural strategies, such as protein-based emulsions or coacervates, improve the stability and delivery of encapsulated micronutrients, indicating the model’s sensitivity to modified food structure design.

Dairy bioactives show clear matrix- and component-dependent digestive stability and functionality under INFOGEST application. Probiotic survival is generally enhanced by structured or solid matrices (e.g., bigels, cheese) compared to liquid systems [24,32], although responses remain strain-specific, and many lactic acid bacteria display intrinsic tolerance to digestion [33]. Functional activity can also be retained, as probiotic-fortified digesta exhibit anti-inflammatory effects in intestinal models. In contrast, extracellular vesicles show partial sensitivity, with reduced marker intensity and particle numbers despite relative preservation of structure and miRNA cargo [55,59]. For other bioactives, such as phenolics, dairy matrices can improve bioaccessibility (up to ~130%) but reduce compound diversity [87]. Overall, the in vitro digestion model effectively captures trends in bioactive protection and release but remains limited in resolving their full structural integrity and physiological relevance.

### 4.5. Concerns: Applications and Downstream Models

The use of the in vitro digestion model, despite the publication of the harmonised recommendation, continues to be coupled with substantial alterations. The area of dairy food is not left out. Such modifications include a lower enzyme-to-food ratio, a higher food/simulated fluid ratio, inconsistent use of gastric lipase, and incubation times longer than the recommended figures [34,38,55,72]. Similarly, there is a growing adaptation of the static digestive model to integrate properties of a dynamic one. The increased availability of instrumentation (e.g., pH-stats, pumps, and jacketed vessels), which can permit the modulation of the biophysical aspects of the digestive model, including pH and temperature, may promote a more prominent widespread adoption of this mode of in vitro digestion. Such measures have been employed and have aided in demystifying the digestion of the lipid component of dairy matrices and associated components (e.g., beta-carotene) [26,54,56]. Similarly, despite the increasing adoption of cell-based approaches alongside the INFOGEST protocol, their implementation remains limited (~20% of the studies). The majority of bioactive functional studies are limited to peptides, while other components (lipids, extracellular vesicles and their cargoes, probiotics) are rarely ventured into. Particularly, the predominance of Caco-2-based models highlights a methodological bottleneck in current INFOGEST applications, where advances in digestion simulation are not matched by equally sophisticated biological validation systems. This overreliance constrains the physiological relevance of post-digestion assessments, as it fails to capture the multicellular, immune, and microbiota-driven processes that influence nutrient bioavailability and bioactivity in vivo. There are also cases of overextension of bioaccessibility into bioavailability when cell absorption assays were employed. All the same, this integrative approach has significantly expanded the functional interpretation of in vitro digestion outcomes. For instance, Tenenbaum et al. [44] work showed a methodological prowess in this regard by combining Caco 2 cells with mucus-secreting and enteroendocrine cells, which allows for a structural and functional setup of the intestine. A few others employed co-cultured cell lines [21] and considered brush border membrane peptidases to aid near-in vivo hydrolysis of peptides in a way that did not influence the digest’s peptide profile [32]. Hence, there is a significant attempt in the evaluation of dairy nutrients’ bioaccessibility via this route. However, care should be taken not to overrepresent the translational relevance of the current post-digestion biological validation systems to the in vivo context. For instance, nutrient permeability in a model lacking physiological diversity [29,32] or the biological activity of the digests on an isolated model [21,40] may not accurately present such functional relevance of digestion products, especially on a system-wide level. Consequently, the heterogeneity observed across studies made it methodologically inappropriate to perform a meta-analysis. Even though the standardised digestion protocol (INFOGEST 2.0) was used.

## 5. Future Research Directions

While INFOGEST-based digestomics is a relatively recent field, the methodological quality of the retrieved studies was robust enough to allow for a detailed systematic analysis and the formulation of evidence-based conclusions. Nevertheless, as the field of food and nutrition science evolves, several avenues open up for expanding the protocol’s application and impact on the milk industrial process [88]. For instance, downstream analysis of the digestion products is mainly focused on compositional and structural examinations. Similarly, a fraction of the literature that endeavoured functional studies employed narrow post-digestion biological systems. Hence, this limits the understanding of the biological activity of bioactive components of dairy products post-digestion [88,89]. To this end, considerations should be given to the use of more dynamic, physiological diverse, and encompassing models such as organoids and microfluidic complex models (e.g., intestine-on-chips) which replicates, more physiologically, the in vivo intestinal wall through the facilitation of cell–cell interactions and the provision of crucial biophysical cues such as perfusion and mechanical actuation and other factors that are vital to the investigation of intestinal epithelial integrity [90,91,92]. Such systems may be extended to include the gut microbiota in a way that permits monitoring microbiome-related changes in the digesta [93,94]. This will change the landscape of study outcome from bioaccessibility (which the current trend mainly depicts) to a more reliable “pseudo-bioavailability”.

**Figure 4:**
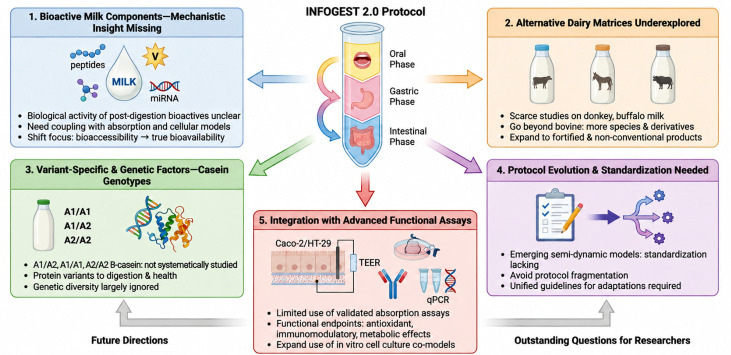
INFOGEST 2.0 in dairy research: Key gaps and priority future directions.

Despite the widespread consumption of buffalo milk in developing regions, its investigation using the INFOGEST protocol remains limited, and this only gives an indication of the predominant use of bovine matrix. This discrepancy likely arises from the technical and economic demands associated with standardised digestion models and advanced analytical techniques. However, several cost-effective approaches, including tiered implementation of the digestion protocol (for example, stratifying the protocol into low-cost core digestion and sophisticated advanced-level models), the use of spectrophotometric and electrophoretic proxies for digestion assessment (low-cost analytics), and collaborative analytical frameworks, could facilitate broader adoption. This will ensure that standardised in vitro digestion models are applied to nutritionally and regionally relevant food matrices, in a manner that not only improves the global applicability of digestion research but also aid regional-relevant policy making. Also, expanding its application to non-conventional dairy matrices and fortified products would also support the development of functional foods, as the current trend indicates an uneven, concentrated effort on bovine and caprine products. Additionally, while significant strides have been made in the examination of dairy lipids’ post-digestive attributes, there is a dearth of inquiries into their functional or biological properties. Hence, replicating functional investigations, at least as seen in the case of dairy proteins, will benefit the fields of food science, nutrition, and health at large. Furthermore, while adaptations of the protocol into semi-dynamic ones, which could provide more comprehensive insights into the temporal aspects of digestion and nutrient release, begin to ensue, there may be an impending problem of “reverse standardisation” of the protocol, where the already-unified protocol becomes a source of varying, instrument-driven, uncoordinated, and therefore, non-replicable models. Hence, a unified recommendation for the semi-dynamic adaptation of the INFOGEST 2.0 model is needed. Such an approach should retain a standardised static core while allowing for the controlled modification of key physiological parameters, including pH progression, enzyme delivery, and gastric emptying. The adoption of predefined dynamic modules, combined with a minimum reporting checklist, would enhance reproducibility and enable meaningful cross-study comparisons while accommodating varying levels of technical capability (Figure 4).

## 6. Conclusions

The INFOGEST protocol has established itself as a robust and internationally harmonised framework for the in vitro investigation of gastrointestinal digestion, with its application to milk and dairy products proving particularly fruitful. This review demonstrates that, since the publication of INFOGEST 2.0, there has been a marked acceleration in the use of the protocol across diverse dairy matrices to interrogate the digestibility of macronutrients, the bioaccessibility of micronutrients, and the generation of bioactive peptides with antioxidant, anti-inflammatory, and antihypertensive potential. The integration of advanced analytical platforms, alongside intestinal epithelial cell models, has substantially laid a meaningful foundation for the mechanistic and functional interpretation of dairy protein and lipid digestion. However, its application remains marked by growing methodological divergence, particularly through protocol modifications and semi-dynamic adaptations that risk undermining reproducibility. Concomitantly, the limited integration of advanced biological validation systems constrains the translation of digestion outcomes into meaningful physiological relevance. Future progress will depend on balancing standardisation with controlled flexibility, through harmonised dynamic extensions, and expanding downstream models toward more physiologically representative systems. Broadening accessibility and application to diverse dairy matrices will also be essential to ensure global relevance. Collectively, these efforts will shift the field from descriptive bioaccessibility toward a more integrated and predictive understanding of nutrient bioavailability.

## Figures and Tables

**Table 1:** Study reporting analytical technique methodology used post INFOGEST on milk and milk products, thematic area, and type of food.

Author/Year	Thematic Area	Analytical Technique	Type of Food
Bottani et al., 2020 [21]	Food Science and Functional Nutrition	UPLC-HR-MS/MS, MTT, Osmolarity,	Bovine milk
Egger et al., 2021 [22]	Food Science	HPLC, GC-MS	Raclette cheese; pasteurised, skimmed and powdered milks
Lin et al., 2021 [23]	Human Health and Nutrition Sciences; Food Sciences	GC	Skimmed milk; cream; bovine milk fat; whole milk
Bollom et al., 2021 [24]	Food Science and Nutrition	GC	Reduced-fat milk (2%) bigels
Barbosa et al., 2021 [25]	Food Science and Agro-industrial Technology	Spectrophotometry	Whey protein
Pinho et al., 2021 [26]	Food Chemistry, Physiology and Digestion	GC-FID, Spectrophotometry, ORAC, SPE	Bovine milk (UHT, whole)
Miralles et al., 2021 [27]	Food Chemistry and Biochemistry	SDS-PAGE, Maldi-TOF/TOF, HPLC-MS/MS, ICP/MS	Bovine milk
Jensen et al., 2021 [28]	Nutrition	LC-ESI-MS/MS; SPE;	Cheese
Paixao-Teixeira et al., 2022 [29]	Physiology and Biochemistry of Animal Nutrition	HPLC	Cheese; cow milk; goat milk
García-Casas et al., 2022 [30]	Food Science and Nutrition	ABTSA, DPPH; FRAP; Folin–Ciocalteu; GC; SDS-PAGE; FAAS	Colada (Whey-based beverage)
Leeuwendaal et al., 2022 [31]	Food Microbiology	PFGE; NMR	Cheddar cheese
Vivanco-Maroto et al., 2022 [32]	Food Science and Nutrition	HPLC-MS/MS; Cell culture	Bovine milk
Tarique et al., 2022 [33]	Food Science	PCR; ELISA	Yoghurt
Sheng et al., 2022 [34]	Food Science and Nutrition	LC-ESI/MS; MS; SDS-PAGE; AEC	Bovine, Danish-cow milks; skimmed milk
Jia et al., 2022 [35]	Food and Biological Engineering	Q-Orbitrap mass spectrometry	Goat milk
Tormási and Abrankò, 2023 [36]	Food Chemistry, Food Technologies, Agriculture and Life Sciences	FTIR-ATR spectroscopy	Sour cream; fat milk; whey; bovine milk; sheep milk; recombined milk
Tormasi and Abrankò, 2023 [37]	Agriculture; Life Sciences	HPLC; GC-FID	Fat milk, cream, and pasta
Sheng et al., 2023 [38]	Food Science, Food Technology and Nutrition Engineering	HPLC; LC-ESI/Q-TOF MS/MS	Skimmed milk; bovine milk; goat milk; breast milk
Lavoisier et al., 2023 [39]	Not Reported	OPA, SDS-PAGE, LC-MS/MS, HPLC	Fermented dairy product and skyr (caseins)
De Espindola et al., 2023 [40]	Food Technology	FLPC; HPLC; RP-HPLC; LC-MS/MS; Cell culture	Whey protein isolate
Du and Jia, 2023 [41]	Food and Biological Engineering	UHPLC-Q-Orbitrap	Goat milk
Du and Jia, 2023 [42]	Food and Biological Engineering	UHPLC-Q-Orbitrap	Goat milk
Rebellato et al., 2023 [43]	Food Safety	ICP-OES; ICP-MS; PCA	Yoghurt
Tenenbaum et al., 2023 [44]	Food Science and Functional Nutrition	SEC-FPLC; qPCR; Cell culture	Bovine milk
Sousa et al., 2023 [45]	Food Science and Nutrition	Kjedhal, SEC, UHPLC, OPA	Whey protein
Bitencourt et al., 2023 [46]	Food Science	Texture analysis, syneresis test, 3D printing evaluation, IDDSI fork test, simulated gastrointestinal digestion, ICP-OES	milk
Reiche et al., 2024 [47]	Agriculture; Life Sciences	MS; HPLC	Milk cows of A1/A2 β-casein genotype
Zelikina et al., 2024 [48]	Biochemistry, Physics	Tensiometry	Whey proteins isolated in chitosan nano-carrier
Muleya et al., 2024 [49]	Nutrition/Dietetics; Agricultural Environmental Sciences	ICP-MS	Bovine skimmed milk
Stefos et al., 2024 [50]	Nutritional Physiology and Feeding; Agricultural Technology	ELISA; qPCR	Whey, yoghurt, bovine milk, sheep milk, goat milk
Costa-Santos et al., 2024 [51]	Food Chemistry, Nutrition	FAAS	Pasteurised whole cow organic milk
Blanco-Doval et al., 2026 [52]*	Food Science	OPA assay; UHPLC-UV (amino acids); SDS-PAGE; peptide fractionation; DIAAS/DIAAR calculations	Mare milk (Basque Mountain Horse) collected from 3 farms, across 6 months of lactation (early, mid, late stages)
Costa-Santos et al., 2024 [53]	Food Chemistry, Nutrition	FAAS, colourimetric phosphorus assay, Kjeldahl protein analysis, OPA assay	A2 milk and conventional milk
Hu et al., 2024 [54]	Food Chemistry	pH-stat for lipid digestion; UV–Vis spectrophotometry (β-carotene); particle size (Mastersizer); ζ-potential; CLSM; FTIR; CD; LF-NMR; rheology	Model dairy-like emulsion systems stabilised by whey protein isolate
Oliver et al., 2024 [55]	Food Science; Nutrition	SDS-PAGE; immunoblotting; nanoparticle tracking analysis (NTA); atomic force microscopy (AFM)	Bovine milk (pooled samples from Holstein Friesian cows)
Comunian et al., 2024 [56]	Food Science	pH-stat lipolysis; GC-FID; particle size analysis; ζ-potential; CLSM	Liquid yoghurt (bovine milk)
Lavoisier et al., 2024 [57]	Food Science	OPA assay; SDS-PAGE; amino acid analysis; GC–MS (FFA/TFA); CLSM	High-protein cream cheese (dairy product) with opposite casein: whey protein ratios (WP-20 = 80:20; WP-80 = 20:80), high fat content
Komatsu et al., 2024 [58]	Health Care and Nutritional Science	SDS-PAGE; SEC;	Human milk and infant formula
Ilori et al., 2025 [59]	Nutrition, Biochemistry	qPCR, Functional analysis	Buffalo milk (extracellular vesicles)
Stork et al., 2025 [60]	Food Science and Technology	Lipid extraction (modified Folch); solid-phase extraction (SPE); GC–MS fatty-acid profiling; PCA statistics	Full-fat cream, full-fat sour cream, cream cheese, and a Gouda-type cheese.
Dalaka et al., 2025 [61]	Food Science, Nutrition	Ultrafiltration (<3 kDa); THP-1 macrophage model; qPCR	Sweet whey (SW) from cheese production: bovine, ovine, caprine, ovine/caprine mix
Gwala et al., 2025 [62]	Food Science	Texture analysis, rheology, tribology, OPA assay, amino acid analysis, SEC-HPLC, confocal laser microscopy	High-protein *dulce de leche* desserts enriched with whey protein isolate (WPI) or WPI + whey protein hydrolysate (WPH)
Stefos et al., 2025 [63]	Food Science; Nutrition	Atomic Absorption Spectrometry (AAS), cell model, qPCR	Yoghurt acid whey (YAW) (bovine, ovine, caprine) vs. full-fat bovine milk
Hueso et al., 2025 [64]	Food Science; Dairy Science	HPLC-ELSD (lipid classes); SDS-PAGE; MALDI-TOF; ABTS; ORAC	Fresh cheese from ultrafiltered cow milk (UFC)
Li et al., 2025 [65]	Nutrition and Food Safety	Caco-2 intestinal absorption model, hCMEC/D3 blood–brain barrier model, nano-LC-MS/MS, molecular docking, BV2 microglial cell assays	Fermented casein hydrolysate
Iwaniak et al., 2025 [66]	Food Science	In silico BIOPEP-UWM analysis, RP-HPLC, LC-MS/MS, ACE inhibition assay, DPP-IV inhibition assay, ABTS, DPPH, FRAP antioxidant assays	Bovine milk protein preparations and their hydrolysates
Dimitrellou et al., 2025 [67]	Food Science and Technology	Viable cell counting (logCFU/g),	Yoghurt
Tellez-Morales et al., 2025 [68]	Biochemical Engineering	Rheology, DSC, FTIR, ABTS assay, DPPH assay, ACE-1 inhibition assay, resistant starch assay	Starch/whey protein isolate (WPI) blends
Thomsen et al., 2026 [69] *	Food Science	OPA assay, LC-ESI/MS Single Q, SDS-PAGE, FPLC purification	Purified bovine caseins
Le Foll et al., 2026 [70] *	Food Science	ICP-OES	Skimmed milk, yoghurt, cheese

* Accepted December 2025, published online in 2026.

**Table 2:** List of compounds studied with the INFOGEST protocol on macronutrients in food matrix.

Author/Year	Compound	Determination	Findings
Bottani et al., 2020 [21]	Proteins	Digestibility	Over 200 caseinophosphopeptides were identified in the digests of hydrolysed, calcium-free, and calcium-bound casein. Such digests exhibit antioxidants comparable to vitamin C in intestinal models.
Egger et al., 2021 [22]	Proteins	Peptide release	Peptide fingerprints are indistinguishable post-intestinal digestion in both static and dynamic IVDs. Higher microbial diversity (via *L. helveticus* presence) in cheeses led to a more diverse peptidome after simulated digestion.
Lin et al., 2021 [23]	Lipids	Lipid’s digestibility	Static and dynamic IVD protocols yielded different milk-fat bioaccessibility (higher with dynamic). However, increasing the food matrix diversity significantly reduced the bioaccessibility in the dynamic model.
Pinho et al., 2021 [26]	Lipids	Bioaccessibility	Employed a semi-dynamic model to analyse gastric oxidative degradation of PUFAs and individual free FAs in conventional and pasture-based milk. There is a higher relative release of medium-chain FA (C8:0–C12:0) than that of longer-chain FA (C14:0–C18:0). Also, the release of selected FAs and PUFAs was higher for pasture-based than conventional milk.
Miralles et al., 2021 [27]	Proteins	Digestibility	Milk casein peptide makeup at the porcine duodenum (in vivo) and gastric endpoint (INFOGEST) is similar. Hence, in vivo and INFOGEST are comparable in their gastric outputs, with a satisfactory correlation coefficient of up to 0.8.
García-Casas et al., 2022 [30]	Proteins, Carbohydrates, Lipids	Digestibility	Fold increase in medium-chain FAs, MUFAs, and PUFAs ranging from 1.07 to 3.71, 1.57 to 4.25, and 1.47 to 5.46 after INFOGEST model on Colada (a whey-based beverage), while SFA drops by approx. half. A total of 251 peptides of 5–25 amino acids were identified after the digestion process; among them, 18 peptides originated from whey proteins (11 peptides from β-casein and 7 from β-lactoglobulin), exhibiting antioxidant activity on Caco-2 cells, while 16 peptides have barley origins.
Sheng et al., 2022 [34]	Proteins (purified κ-casein)	Digestibility	Bovine milk native k-casein variants’ digestibility ranges from 37% to about 40%, as determined by the degree of hydrolysis post-IVD (intestinal phase) in variants A, B, and E, with B being the least. This degree is further improved by the desialylation of the purified forms.
Jia et al., 2022 [35]	Lipids/Phospholipids	Bioaccessibility	Goat milk subjected to homogenization treatment at 30 MPa contained more abundant phospholipids, in particular PC (phosphocholine) and PE (phosphoethanolamine), than raw milk. Homogenization decreased the loss ratio of PC but increased the overall digestibility of goat milk.
Vivanco-Maroto et al., 2022 [32]	Proteins	Bioaccessibility	The adaptation of brush border enzymes to the INFOGEST produced additional cleavage to those observed with pancreatic enzymes in casein and whey proteins, which matched those in human jejunum. Although the peptidic fingerprint/variety remained lower than those found in vivo, this may provide a closer situation to in vivo.
Sousa et al., 2023 [45]	Proteins	Digestibility	Established an analytical workflow which was combined with INFOGEST to determine protein digestibility in whey protein isolate (amongst other products), with 3 measures: total N, primary amines (OPA), and individual AAs. This yielded high correlations between in vitro and in vivo digestibilities and DIAAR (digestible indispensable amino acid ratio) values. WPI exhibited 95 to 99%, compared to 95% in vivo.
Tormási and Abrankò, 2023 [36]	Lipids	Digestibility	Milk fat from sour cream exhibited low digestibility (61.1%, FFA release) when digested using simulated digestion in isolation due to globule flocculation. However, increasing food matrix diversity (in a meal) increased lipolysis (by 9% to 66.8%), potentially due to the flocculation-protecting effect of the proteins from the food matrix.
Sheng et al., 2023 [38]	Proteins	Peptide Release	While the B variant of bovine k-casein showed a slower digestion rate compared to the A and E variants, its desialylation resulted in an increased rate, indicating the need for an understanding of dairy protein from genetic and biochemical points of view.
Reiche et al., 2024 [47]	Proteins	Peptide Release	Milk from A1A1 cows showed a different gastric protein digestion than milk of A1A2 and A2A2 cows. This includes smaller amounts of β-casomorphin (BCM)21-associated peptides and larger amounts of BCM11-associated peptides.
De Espindola et al., 2023 [40]	Proteins	Bioaccessibility	About 35% proteolysis of whey protein was observed, yielding resistant low-molecular-weight peptides. The achieved peptides promoted antioxidant defence in a Caco-2 model through ROS production inhibition and GSH and SOD activation.
Du and Jia, 2023 [41]	Proteins	Peptide Release	Identified a total of 47 dipeptides, 59 tripeptides, and 21 tetrapeptides post-digestion in goat milk, whose content increased with increasing digestion. These short-chain peptides tend to have a Pro residue at the C-terminal or penultimate position, a slightly greater negative charge at pH 7.0, and fewer C-terminal aliphatic and polar amino acids.
Du and Jia, 2023 [42]	Proteins	Peptide Release	Pinpointed 4 novel antihypertensive short-chain peptides from 186 identified in goat milk digesta.
Tenenbaum et al., 2023 [44]	Proteins	Isolated whey protein and hydrolysate effects on glucose homeostasis	Whey protein hydrolysate was more hydrolysed post-digestion than whey protein isolates, and it induced a significantly higher GLP-1 from STC cell models. It also inhibited DPP-IV activity in a Caco-2/HT29-MTX co-culture, indicating possible modes of hypoglycaemic effects.
Tormási and Abrankò, 2023 [37]	Lipids	Short- and medium-chain triglycerides	Increasing milk cream matrix diversity with black tea or grape seed powder resulted in a significant decrease in lipolysis (from 77.1% fatty acid release initially recorded). This observation was substrate dependent, i.e., it did not apply to baked beef. Also noted a decrease in lipolysis in the absence of gastric lipase (to 69.2%).
Blanco-Doval et al., 2026 [52] *	Proteins, amino acids	Digestibility and peptide release	Mare milk proteins exhibited average digestibility (Di) of 94 to 96%, depending on the determination method, after being subjected to the INFOGEST model, although milk at late lactation showed lower Di. Similarly, almost all the amino acids examined have a Di above 90% (except alanine). The DIAARs of the amino acids were also determined.
Komatsu et al., 2024 [58]	Proteins	Digestibility	There was no significant difference in the digestion rates of human milk and infant formula (in vitro digestion rate = approx. 80%).
Prete et al., 2024 [71]	Carbohydrates (exopolysaccharides, EPSs) (Postbiotics)	Quantification of EPSs produced during fermentation; relation with functional activity	In vitro digesta of fermented milk fortified with beneficial lactic-acid bacteria strains showed anti-inflammatory activity in an inflamed intestinal model (NCM460 human cells) through reduced ROS release. This activity positively correlated with EPS production of the lactic-acid bacteria.
Hu et al., 2024 [54]	Proteins (Whey protein isolate)	Structural changes and interfacial behaviour during digestion	Whey protein isolate emulsion improved the loaded beta-carotene bioaccessibility (~55%).
Costa-Santos et al., 2024 [53]	Free amino groups (NH2)	Digestibility	A2 milk released approximately twice as many free amino groups during early intestinal digestion, suggesting improved digestibility
Comunian et al., 2024 [56]	Lipids	Protein and lipid digestibility	Flaxseed oil coacervates were incorporated into yoghurt, yielding a total protein release ranging between 46.6 and 61.2%, with encapsulation ingredients added in free form showing lower cumulative protein release but higher free-fatty-acid release.
Liu et al., 2024 [72]	Proteins	Gastric digestibility and digestion rate	Whey protein isolate-based heat- or acid-induced gels were digested with the INFOGEST model. Homogeneous gels showed the highest in vitro protein gastric digestion rate. Increasing gel surface area enhanced protein digestion depending on the microstructure.
Lavoisier et al., 2024 [57]	Proteins, Lipids	Protein digestibility and proteolysis kinetics	Digesting two high-protein dairy products similar to cream cheese, the degree of proteolysis was lower in the elderly model than in the young adult (−16% for WP-20, and −20% for WP-80). This is similar for the degree of lipolysis in high-casein product, although the high-whey-protein formulation is similar for both models.
Stork et al., 2025 [60]	Lipids	Digestibility and bioaccessibility	The in vitro digestion of high-fat commercial dairy products yielded similar numbers of lipid classes, although the full-fat cream monoacyglycerol class was comparatively less diverse. Some products also showed a more homogenous lipid digestion than others.
Dalaka et al., 2025 [61]	Whey proteins, bioactive peptides (<3 kDa)	Generation of digestion-resistant peptides, immunomodulation activity	Three (3) kDa digest cutoff of sweet whey digest of bovine, ovine, and caprine milk all exhibited anti-inflammatory responses in an LPS-challenged model of macrophages (THP-1).
Hueso et al., 2025 [64]	Proteins, lipids	Digestibility, size and class distribution	Enriching cheese produced from ultrafiltered milk with omega-3 FA (W) or milk fat globule membrane (M) did not negatively affect protein or lipid digestibility, while improving the product’s antioxidant capacity. Peptide relative abundance in the digesta was significantly higher in WM sample (82.6%) than in the control (without; 38.8%); however, the lipolysis rate is similar.
Gwala et al., 2025 [62]	Proteins	Digestibility	Whey protein-enriched desserts can provide soft textures suitable for older adults while maintaining excellent protein digestibility and amino acid availability
Li et al., 2025 [65]	Proteins, peptides	Bioaccessibility, peptides identification	63 peptides were present across all stages.
Iwaniak et al., 2025 [66]	Peptides	Bioactive peptides identified	Digestion released bioactive peptides from milk protein preparations
Tellez-Morales et al., 2025 [68]	Proteins	Biofunctional, rheological, thermal, and structural properties	Extrusion can enhance biofunctional and digestion-related properties of starch–protein food systems
Thomsen et al., 2026 [69] *	Proteins	Digestibility	αs-caseins were more affected by phosphorylation level than β-caseins

* Accepted December 2025, published online in 2026.

**Table 3:** List of compounds studied with the INFOGEST 2.0 protocol on micronutrients and other bioactive components in the food matrix.

Author/Year	Compound	Determination	Findings
Bollom et al., 2021 [24]	Probiotics	Digestibility and stability	A formulated bigel was effective in increasing the survival of milk-fortified probiotics (*Bifidobacterium lactis* and *Lactobacillus acidophilus*) during IVD, although this survival rate is probiotic-dependent.
Jensen et al., 2021 [28]	Vitamin K	Bioaccessibility	Vitamin K bioaccessibility in cheese, as assessed by INFOGEST, ranges from 6 to 80%, in a manner that is not explained by differing cheese products, ripening time, starter culture, fat content, and water content.
Barbosa et al., 2021 [25]	Encapsulated β-carotene	Effect of pH and temperature	Whey protein isolates, forming complex coacervates with carboxymethylcellulose, offer higher stability (83.37%) and acceptable bioaccessibility (31.16%) to encapsulated components such as micronutrients (beta-carotene) and functional foods (sacha inchi oil) during IVD.
Paixao-Teixeira et al., 2022 [29]	Calcium, magnesium and zinc	Bioaccessibility	Mineral bioaccessibility after in vitro digestion of selected Brazilian cheeses was in the range of 44.4–74.7%, 54.8–66.1% and 18.2–38.7% for Ca, Mg and Zn, respectively. Fresh cheese provided the highest soluble Ca and Mg levels after digestion. Zn from matured cheese was efficiently absorbed by Caco-2 cells.
Bitencourt et al., 2023 [46]	Calcium, magnesium, potassium, and phosphorus	Bioaccessibility	Mineral bioaccessibility remained high: calcium >80%, magnesium/potassium/phosphorus >90%. Texture modification did not negatively affect mineral bioaccessibility.
Stefos et al., 2024 [50]	Iron (Fe)	Influence of acidic whey on iron bioaccessibility	Iron (Fe) uptake from milk digesta in Caco-2 cells did not differ from that of Fe-fortified yoghurt acid whey as measured by CaCO_2_ Fe (nmol/ug protein), DCYTB, DMT1, FPN1, and HEPH.
Costa-Santos et al., 2024 [51]	Calcium, magnesium, sodium (Na), phosphorus (P) and potassium (K)	Bioaccessibility	Organic (ORG) milk samples showed protein content equivalent to conventional (CNV) and lower lactose and lipid content. Mg had the best bioaccessibility in both samples (> 60%). ORG showed the highest values of bioaccessible calcium (12–65%) against CNV (11–27%), while phosphorus was higher (74%) in CNV.
Muleya et al., 2024 [49]	Calcium (Ca)	Bioaccessibility in bovine vs. various plant-based milks	Combining isotopically labelled calcium (^43^Ca) with INFOGEST, several plant foods with higher calcium bioaccessibility than skimmed milk (29.9%) were identified. However, only 3 (kale, finger millet and fortified white bread) were identified as good sources of Ca, with kale providing 5 x more bioaccessible Ca than skimmed milk.
Rebellato et al., 2023 [43]	Calcium; potassium; magnesium; sodium; phosphorus; iron; copper (Cu); manganese (Mn); zinc; selenium (Se); chromium (Cr); cobalt (Co); molybdenum (Mo); aluminium (Al); barium (Ba); nickel (Ni)	Bioaccessibility	The bioaccessible Ca was higher in animal-based yoghurt (41%) than in plant yoghurt. Molybdenum bioaccessibility was very high at 71%.
Oliver et al., 2024 [55]	Milk extracellular vesicles (EVs)	Stability, integrity and recovery of EVs after in vitro digestion	The in vitro digestion employed reduced the concentration and the intensity of selected markers (TSG101 and CD9) of the EVs in bovine milk. Although experimental design entailed reduced incubation time and a high food/simulated fluid ratio.
Hu et al., 2024 [54]	β-Carotene (provitamin A)	Bioaccessibility, recovery and stability after in vitro digestion	Whey protein isolate emulsion improved the loaded beta-carotene bioaccessibility (by approx. 55%).
Costa-Santos et al., 2024 [53]	Calcium, magnesium, phosphorus, sodium, potassium	Bioaccessibility	A2 milk showed similar mineral composition and mineral bioaccessibility to non-A2 milk
Ilori et al., 2025 [59]	Conserved microRNAs	Stability	Buffalo milk showed no structural differences in its EVs and selected miRNA cargo after IVD, besides a slight reduction in particle numerosity. Hence, they have the potential to influence our biology at a global level, acting as regulators of the nervous and immune systems.
Stefos et al., 2025 [63]	Calcium	Bioaccessibility (soluble fraction)	Calcium bioaccessibility of yoghurt acid whey (YAW; 41%) was significantly higher than that of milk (17.7%), as determined through INFOGEST. However, Ca uptake, through a Caco-2 model, as well as the transcription of Ca absorption-related genes (VDR, TRPV6, S100G, and PMCA1), was comparable for both products.
Dimitrellou et al., 2025 [67]	Probiotics	Viability	Apple fibre-enriched yoghurt may serve as an effective synbiotic functional food with enhanced probiotic delivery through the gastrointestinal tract
Le Foll et al., 2026 [70] *	Calcium	Solubility, Bioaccessibility	The calcium bioaccessibility of dairy matrices (yoghurt, cheese, milk) was particularly high (between 19 and 34%). However, calcium bioaccessibility was more disparate for plant-based dairy alternatives (between 5 and 20%) and whole plant foods (between 1 and 27%).

* Accepted December 2025, published online in 2026.

**Table 4:** List of studies using the INFOGEST 2.0 protocol on cell cultures.

Author, Reference	Model	Determination
Bottani et al., 2020 [21]	Caco-2/HT-29, Saos-2	Antioxidant activity
García-Casas et al., 2022 [30]	Caco-2	Antioxidant activity
Paixao-Teixeira et al., 2022 [29]	Caco-2	Transepithelial absorption and cellular uptake were evaluated, and cell viability was assessed to exclude cytotoxic effects.
Vivanco-Maroto et al., 2022 [32]	Caco-2	Transepithelial transport and apparent permeability (Papp) of peptides; epithelial integrity monitored by TEER and Lucifer Yellow assay.
De Espindola et al., 2023 [40]	Caco-2	Antioxidant activity
Tenenbaum et al., 2023 [44]	Caco-2; HT29-MTX; STC-1	Hypoglycaemic potential
Dalaka et al., 2025 [61]	THP-1	Modulation of inflammation-related gene expression (qPCR)
Stefos et al., 2025 [63]	Caco-2	Bioaccessibility (cell transport)
Li et al., 2025 [65]	Caco-2, LPS-induced BV2 microglial cells	Method to identify bioaccessible and bioactive peptides from fermented dairy products

## Data Availability

No new data were created or analysed in this study. Data sharing is not applicable to this article.

## Abbreviations

ABAP	2,2′-azobis(2-amidinopropane) dihydrochloride
AEC	Anion exchange chromatography
DCYTB	Duodenal cytochrome gene
DMT1	Divalent metal transporter 1
DPP-IV	Dipeptidyl peptidase IV
ELISA	Enzyme-Linked Immunosorbent Assay
FAAS	Flame atomic absorption spectroscopy
FPN1	Ferroportin 1
FTIR-ATR	Fourier Transform Infrared–Attenuated Total Reflection
GC	Gas Chromatography
GC-FID	Gas Chromatography with Flame Ionisation Detector
GCG	Glucagon
GLP-1	Glucagon-like peptide 1
HEPH	Hephaestin
HPLC	High-Performance Liquid Chromatography
ICP-MS	Inductively Coupled Plasma–Mass Spectrometry
IFN	Interferon
IL	Interleukin
IVD	In vitro digestion
LC/MS	Liquid Chromatography/Mass Spectrometry
LC-ESI	Liquid Chromatography–Electrospray Ionisation
MS/MS	Tandem mass spectrometry.
MS	Mass Spectrometry
MUFA, PUFA, SFA	Mono-unsaturated, poly-unsaturated, and saturated fatty acids
NMR	Nuclear Magnetic Resonance
OPA	Ortho Phthalic Aldehyde
PCSK1	Proprotein convertase subtilisin/Kexin type 1
PMCA1	Plasma membrane calcium-transporting ATPase-1
qPCR	Quantitative Polymerase Chain Reaction
Q-TOF	Quadrupole–Time of Flight
ROS	Reactive oxygen species
S100G	S100 calcium-binding protein
SDS-PAGE	Sodium Dodecyl Sulphate Poly Acrylamide Gel Electrophoresis.
TEER	Trans-epithelial electrical resistance
TNF	Tumour necrosis factor
TRPV6	Transient receptor potential vanilloid membrane 6
UHPLC-Q	Ultra-High-Performance Liquid Chromatography–Quadrupole
VDR	Vitamin D receptor
